# TAF1 Suppresses MHC-I Expression and Correlates with Poor Immunotherapy Response in Small Cell Lung Cancer

**DOI:** 10.3390/biomedicines14050973

**Published:** 2026-04-23

**Authors:** Qing Gao, Kehong Wei, Deshen Pan, Yufei Xi, Chaoliang Xu, Deshui Jia

**Affiliations:** 1 Department of Radiation Oncology, Institute of Clinical Research, Shanghai General Hospital, Shanghai Jiao Tong University School of Medicine, Shanghai 200080, China; gaoqing1525@163.com; 2 Department of Thoracic Surgery, Shanghai General Hospital, Shanghai Jiao Tong University School of Medicine, Shanghai 200080, China; weikh99@163.com (K.W.); deshenpan0914@163.com (D.P.); xiyufei1016@163.com (Y.X.)

**Keywords:** small cell lung cancer, epigenetic regulator, TAF1, immunotherapy, antigen presentation

## Abstract

**Background:** Small cell lung cancer (SCLC) is an aggressive neuroendocrine tumor characterized by an intrinsic resistance to immunotherapy, primarily due to its low immunogenicity and immune-cold tumor microenvironment. The mechanisms underlying this resistance remain poorly understood. **Methods:** A systematic screen of 796 epigenetic regulators was performed to identify candidate genes associated with effector CD8^+^ T cell infiltration and clinical outcomes following chemoimmunotherapy in SCLC. This analysis integrated several public SCLC datasets, including single-cell RNA sequencing (scRNA-seq) data from 20 SCLC tumors, bulk RNA-seq data from the IMpower133 cohort, proteomic profiles from the TU-SCLC cohort, and an independent scRNA-seq dataset of 39 SCLC tumors. In vitro and in vivo functional experiments were conducted to investigate the role of the candidate genes in SCLC. **Results:** The epigenetic regulator TAF1 emerged as a key candidate, with its expression negatively correlating with effector CD8^+^ T cell infiltration in SCLC. Clinically, patients with low *TAF1* expression in tumors showed better outcomes following atezolizumab-based chemoimmunotherapy, particularly in *ASCL1*-high tumors. Additionally, *TAF1* expression was inversely correlated with MHC-I expression. Knockdown of *TAF1* in SCLC cells restored MHC-I expression, suppressed tumor growth in immunocompetent mice, and increased CD8^+^ T cell infiltration. **Conclusions:** TAF1 functions as a potential epigenetic suppressor of MHC-I expression in SCLC. Targeting TAF1 may represent a promising therapeutic strategy to enhance immunotherapy efficacy in SCLC.

## 1. Introduction

Small cell lung cancer (SCLC) is an aggressive neuroendocrine (NE) malignancy characterized by rapid growth, early metastasis, and poor prognosis, despite initial chemosensitivity [1]. Platinum-based chemotherapy combined with immune checkpoint inhibitors (ICIs) targeting PD-L1 has been approved as first-line therapy for extensive-stage SCLC [2]. However, the clinical benefit of ICIs remains limited and highly heterogeneous across patients, reflecting the profound immunological diversity of SCLC [3,4]. Identifying the determinants of antitumor immune responses and predictive biomarkers is therefore critical for optimizing immunotherapeutic strategies.

Epigenetic dysregulation has emerged as a hallmark of cancer biology [5]. Comprehensive genomic analyses have revealed frequent inactivation of key epigenetic regulators, including CREBBP and EP300, in SCLC [6,7]. Notably, targeting epigenetic regulators such as EZH2 and LSD1 (KDM1A) has been shown to potentiate immunotherapy response in preclinical models of SCLC [8,9,10]. Currently, several clinical trials are testing combinations of EZH2 inhibitors with immunotherapy in SCLC (NCT06807632; NCT05353439). These findings underscore the critical roles of epigenetic regulation in shaping immunotherapy responses in SCLC.

TAF1 (TATA-box binding protein-associated factor 1), the largest subunit of the TFIID complex, is a multifunctional histone acetyltransferase that regulates transcription initiation, chromatin states, and cell-cycle progression [11,12]. The oncogenic role of TAF1 has been reported in several cancer types [13,14,15]. TAF1 inhibition has been shown to induce endogenous retrovirus expression, activate IFN signaling, and suppress growth in a subset of triple-negative breast cancer, highlighting its potential role in modulating tumor immunogenicity [16]. Moreover, a recent study demonstrated that TAF1 degradation activates p53 signaling and induces apoptosis in acute myeloid leukemia, further supporting its therapeutic potential [17]. However, the role of TAF1 in SCLC, especially in regulating tumor immunogenicity and immunotherapy response, remains largely unexplored.

In this study, through integrative analysis of multiple public SCLC datasets, we identify TAF1 as an epigenetic regulator inversely associated with CD8^+^ T cell infiltration and major histocompatibility complex class I (MHC-I) expression. Functionally, we demonstrate that TAF1 depletion restores MHC-I expression and suppresses tumor growth in SCLC models. Together, this study identifies TAF1 as a potential epigenetic driver and a promising therapeutic target to enhance immunotherapy efficacy in SCLC.

## 2. Materials and Methods

### 2.1. Study Cohorts and Data Sources

ScRNA-seq data of 20 SCLC tumors analyzed in this study were obtained from a previously published dataset [18]. Proteomic and survival data for SCLC patients were obtained from the TU-SCLC cohort [19], while transcriptomic and survival data were derived from the IMpower133 cohort [3,4]. An independent scRNA-seq dataset comprising 39 SCLC samples [20] was used for external validation.

### 2.2. Re-Analysis of scRNA-Seq Data

Raw unique molecular identifier (UMI) count matrices were quality-controlled and normalized using the Seurat (version 4.4.0) R package. Cells with fewer than 300 expressed genes or with >10% mitochondrial transcripts were excluded. Gene expression matrices were log-normalized using the NormalizeData function and scaled using ScaleData. Highly variable genes (*n* = 2000) were identified via the FindVariableFeatures function.

Principal component analysis (PCA) was performed on the top variable genes, followed by batch correction across samples using Harmony (version 1.2.3) to minimize inter-sample variation. The first 13 principal components were applied for cell clustering using the FindNeighbors and FindClusters functions with a resolution of 0.1. Dimensional reduction visualization was achieved with the RunUMAP function.

### 2.3. Cell Type Annotation and T Cell Trajectory Analysis

Major cell types were annotated based on canonical marker genes collected from published signatures, including *EPCAM*, *CHGA*, and *INSM1* for SCLC cells; *PTPRC*, *CD3E* for T cells; and *CD68*, *S100A8* for monocyte macrophages (Mono/macro). Sub-clustering of T and NK cells was performed using Seurat and independently verified using Scanpy (version 1.9.8) to ensure cross-platform consistency. For T cell differentiation trajectory analysis, Palantir (1.3.2) and CellRank (2.0.2) algorithms were used to infer pseudotime dynamics and terminal cell states. Gene expression dynamics across pseudotime were further visualized using CellRank.

### 2.4. Identification of Candidate Genes Associated with CD8^+^ T Cell Infiltration and Overall Survival

A total of 796 known epigenetic modifiers [21,22] were screened for candidate genes across multiple SCLC datasets. For the scRNA-seq cohort (20 SCLC tumors, Rudin et al.) [18], Pearson correlation was performed between the expression of 796 epigenetic regulators in tumor cells and the proportion of effector CD8^+^ T cells among total T cells. Genes with a correlation coefficient (*R*) < 0, *p*-value < 0.05, FDR < 0.15, and power > 0.8 were selected, and the results are presented in Table S1.

For the IMpower133 transcriptomic cohort (271 patients), patients were stratified by median expression of each gene. Univariate Cox regression was performed to compare overall survival (OS) between the atezolizumab and placebo arms within the low-expression group (gene expression < median, atezo vs. placebo). Genes with hazard ratio (HR) < 1 and *p*-value < 0.05 were selected as candidates, with lower expression predicting better response to immunotherapy. Results are presented in Table S2.

For the TU-SCLC proteomic cohort (112 patients), optimal expression cutoffs for each gene were determined using maximally selected rank statistics. Patients were stratified into high- and low-expression groups based on the best cutoff, and OS was compared using univariate Cox proportional hazards regression. Genes with HR < 1 and *p* < 0.05 were considered protective, with lower expression associated with better survival. Results are presented in Table S3. For all analyses across the three cohorts, Benjamini–Hochberg FDR correction was applied. Post hoc power analysis was performed using the pwr package (1.3.0) in R.

To evaluate the independent predictive value of *TAF1* expression in the IMpower133 cohort, multivariate Cox proportional hazards regression was performed within *TAF1*-low and *TAF1*-high subgroups (stratified by median expression). This analysis adjusted for the following covariates: age (<65 vs. ≥65 years), sex, number of metastatic sites, brain metastasis, liver metastasis, ECOG performance status, LDH level (≤ULN vs. >ULN), blood-based tumor mutational burden (bTMB), and PD-L1 IC score. Results are presented as forest plots with HRs and 95% confidence interval (CI).

### 2.5. Animal Experiments

*Rb1*^f/f^;*Trp53*^f/f^ (RP) mice were generated by crossing *Rb1*^f/f^;*Trp53*^f/f^;*Myc*^LSL/LSL^ mice (RPM, #029971) with C57BL/6 mice. RPM mice were obtained from The Jackson Laboratory (Bar Harbor, ME, USA), and C57BL/6 mice (SM-001) were obtained from the Shanghai Model Organisms Center (Shanghai, China). All mice were housed under specific pathogen-free (SPF) conditions. To develop a genetically engineered mouse model of SCLC, RP mice were infected with Ad-CMV-Cre viruses as we previously described [23].

For subcutaneous allograft tumor models, 1.0 × 10^6^ cells resuspended in 100 μL of PBS were injected subcutaneously into the right flank of 6–8-week-old C57BL/6 mice. Tumor growth was monitored every three days using caliper measurements, and tumor volume was calculated using the formula: volume = 0.5 × length × width^2^. At the experimental endpoint, mice were euthanized, and tumors were harvested for subsequent flow cytometry analysis.

### 2.6. Cell Culture

Primary mouse SCLC cell line RP (ASCL1-high subtype) was generated from primary SCLC tumors derived from an RP mouse. Human SCLC cell lines NCI-H146 (HTB-173) and DMS53 (CRL-2062) were obtained from American Type Culture Collection (ATCC, Manassas, VA, USA). 293T cell line was obtained from the Cell Bank of the Chinese Academy of Sciences (Shanghai, China). RP cell line was cultured in DMEM medium with 15% FBS. DMS53 and 293T cell lines were cultured in DMEM with 10% FBS. NCI-H146 was cultured in RPMI 1640 medium with 10% FBS; all media were supplemented with 100 U/mL penicillin and 100 µg/mL streptomycin at 37 °C in 5% CO_2_.

### 2.7. Lentiviral Vector Construction and Viral Infection

The pLKO.1-TRC cloning vector (#10878, Addgene, Watertown, MA, USA) was a gift from David Root. The psPAX2 (#12260, Addgene) and pMD2.G (#12259, Addgene) vectors were provided by Didier Trono. shRNA oligonucleotides targeting the mouse *Taf1* gene (5′-TTCTATCTTGGAGTCTATTAT-3′), the human *TAF1* gene (5′-GCTGCAAGCATTTGAGAACAA-3′), and a non-targeting control (5′- CCTAAGGTTAAGTCGCCCTCG-3′) were subcloned into the pLKO.1-TRC vector using standard protocols. Lentivirus was produced in HEK 293T cells using Lipofectamine 3000 (L3000015, ThermoFisher, Waltham, MA, USA), by co-transfecting pLKO.1 vectors with the psPAX2 and pMD2.G packaging plasmids. Viral supernatants were collected 48 h post-transfection, filtered (0.45 µm), and used to infect target cells in the presence of 8 µg/mL polybrene (Sigma, St. Louis, MO, USA). Infected cells were selected with 2 µg/mL puromycin for 5–7 days. Knockdown efficiency was confirmed by Western blotting.

### 2.8. Western Blotting

Western blotting was performed using standard protocols. Briefly, tumor cells were harvested and lysed in T-PER Tissue Protein Extraction Reagent (78510, ThermoFisher) supplemented with protease and phosphatase inhibitors. Protein lysates (10–20 µg) were separated via SDS-PAGE and transferred onto nitrocellulose membranes. Membranes were blocked in 5% non-fat milk in TBST for 1 h at room temperature, followed by overnight incubation at 4 °C with primary antibodies: Anti TAF1 (Proteintech, Rosemont, IL, USA, Cat No. 20260-1-AP; 1:1000, Rabbit) and anti-β-Actin (Service-bio, Wuhan, China, Cat No. GB15003; 1:3000, Rabbit). After washing, membranes were incubated with HRP-conjugated Rabbit IgG secondary antibody for 1 h at room temperature. Band intensities were detected using SuperSignal West Pico Chemiluminescent Substrate (34580, ThermoFisher) and imaged using a FluorChem E system (ProteinSimple, San Jose, CA, USA).

### 2.9. Quantitative PCR Analysis

Total RNA was isolated from tumor cells with TRIzol reagent (15596026CN, ThermoFisher), followed by cDNA synthesis using HiScript III All-in-one RT SuperMix (R333-01, Vazyme, Nanjing, China). qPCR was conducted with ChamQ Universal SYBR qPCR Master Mix (Q711-02, Vazyme), and primer sequences are listed in Table S4. The experiment was performed in three independent replicates.

### 2.10. Flow Cytometry

Approximately 150 mg of allograft tumor tissues from each tumor were collected for single-cell suspension preparation. Tumors were minced into 1 mm^3^ fragments, and digested with a collagenase (Sigma-Aldrich, St. Louis, MO, USA, #C5138)/hyaluronidase (Sigma-Aldrich, #H3506)/DNase I (Sigma-Aldrich, #DN25) enzyme mixture at 37 °C for 1 h with agitation. Single-cell suspensions were obtained by filtering through a 70 μm strainer and washing twice with PBS. A total of 1–2 × 10^6^ cells per sample were first stained for viability with Zombie Aqua Fixable Viability Dye (BioLegend, San Diego, CA, USA, #423102) and then blocked with Mouse BD Fc Block (BD Biosciences, San Jose, CA, USA, #553141). Surface staining was performed using the following antibody panel: PerCP/Cyanine5.5 anti-mouse CD45 (#103132), Alexa Fluor 700 anti-mouse/human CD11b (#101222), Alexa Fluor 700 anti-mouse CD19 (#115527), Alexa Fluor 700 anti-mouse NK 1.1 (#108730), FITC anti-mouse CD3 (#100203), APC/Fire 750 anti-mouse CD4 (#100459), and PE/Cyanine7 anti-mouse CD8a (#100722), all from BioLegend. Samples were acquired on a BD LSRFortessa cytometer (BD Biosciences) and analyzed with FlowJo software (10.8.1).

For in vitro cultured SCLC cell lines, 1–2 × 10^6^ cells were stained with 7 AAD Viability Staining Solution (BioLegend, #420404) together with either APC anti-human HLA A, B, C (BioLegend, #311409; for human lines) or APC anti-mouse H-2Kb/H-2Db (BioLegend, #114614; for mouse lines). An APC Mouse IgG2a, κ Isotype control (BioLegend, #400220) served as the negative control. Data were collected on a BD Accuri C6 Plus flow cytometer (BD Biosciences) and processed using FlowJo software. All experiments were performed in three independent replicates.

### 2.11. Cell Viability Assay

Cell viability was assessed with the CellTiter-Glo Luminescent Cell Viability Assay Kit (Promega, Madison, WI, USA, #G7571) according to the manufacturer’s protocol. In brief, cells were seeded at 3000 cells per well in 100 μL of culture medium into white 96-well plates and cultured for up to 7 days. At indicated time points, 100 μL of CellTiter-Glo reagent was added to each well. Luminescence signals were measured using a BioTek Synergy H1 microplate reader (Winooski, VT, USA). All experiments were performed in three independent replicates.

### 2.12. Statistical Analysis

Growth curves from in vitro and in vivo experiments were analyzed using two-way ANOVA, with data presented as mean ± standard error of the mean (s.e.m.). Data from all other experiments were analyzed using unpaired two-tailed Student’s *t*-tests, and results are reported as mean ± standard deviation (s.d.). Pearson correlation analysis was used to assess associations between continuous variables. Univariate and multivariate regression analyses were used to investigate the correlations between variables and clinical outcomes. Survival outcomes were compared using Kaplan–Meier curves and the log-rank test. Statistical significance was defined as *p* < 0.05. All analyses were conducted using R (version 4.5.1) and Python (version 3.9).

## 3. Results

### 3.1. Re-Analysis of Public SCLC ScRNA-Seq Data

**Figure 1:**
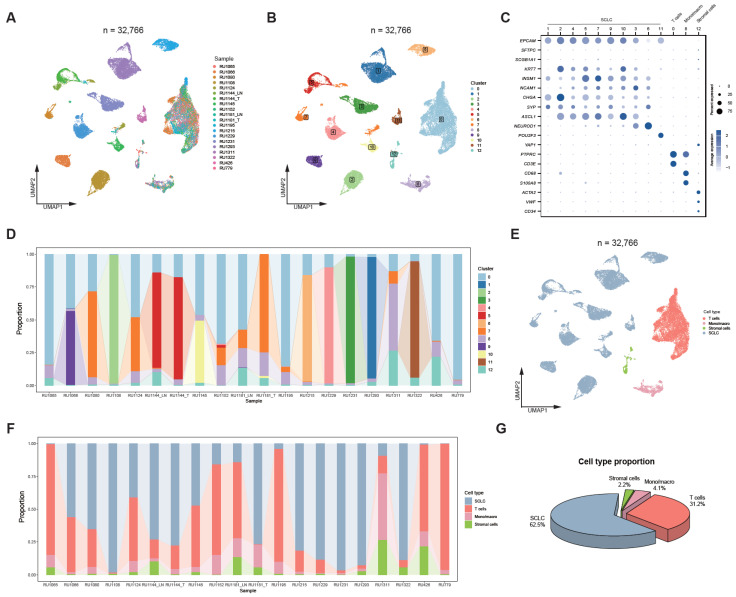
Re-analysis of public SCLC scRNA-seq data. (**A**) UMAP visualization of all cells, colored by sample identity, from 20 SCLC samples in the Rudin et al. cohort [18]; (**B**) UMAP plot showing all cells colored by cell clusters; (**C**) Dot plot showing the expression of representative marker genes across cell types; the scale bar indicates average gene expression; dot sizes represent the proportion of cells expressing each gene; (**D**) Stacked bar plots showing the proportions of distinct cell clusters across samples; (**E**) UMAP visualization of all cells colored by major cell types; (**F**) Stacked bar plot showing the proportional distribution of major cell types across samples; (**G**) Pie chart showing the proportions of individual cell types across the 20 SCLC samples.

To delineate the immune cell landscape of SCLC, we re-analyzed scRNA-seq data from 20 SCLC tumors in the Rudin et al. cohort [18]. Following quality control, normalization, and dimensionality reduction, a total of 32,766 high-quality cells were retained for downstream analysis (Figure 1A). Unsupervised clustering identified 13 distinct cellular clusters, which could be broadly categorized into tumor cells, T cells, monocytes/macrophages (Mono/macro), and stromal cells (Figure 1B–E and Figure S1A).

Within the malignant compartment, we observed significant transcriptional heterogeneity among tumor cells. The majority of tumor cells exhibited *ASCL1* expression, indicating an SCLC-A subtype identity (Figure 1B,C). In contrast, Cluster 6 displayed strong *NEUROD1* expression, representing the SCLC-N subtype, which was predominantly derived from sample RU1215 (Figure 1C,D). Additionally, Cluster 11 showed specific expression of *POU2F3*, corresponding to the SCLC-P subtype, and was enriched in sample RU1322 (Figure 1C,D).

Regarding immune cell populations, the proportion of immune cells was relatively low in more than half of the tumors, reflecting the characteristic immune-cold tumor microenvironment (TME) of SCLC (Figure 1F,G). However, most tumors still contained detectable numbers of T cells, with some tumors showing a predominant T cell population, providing a basis for more detailed T cell profiling in subsequent analyses.

### 3.2. T Cell Heterogeneity and Evolution in SCLC

**Figure 2:**
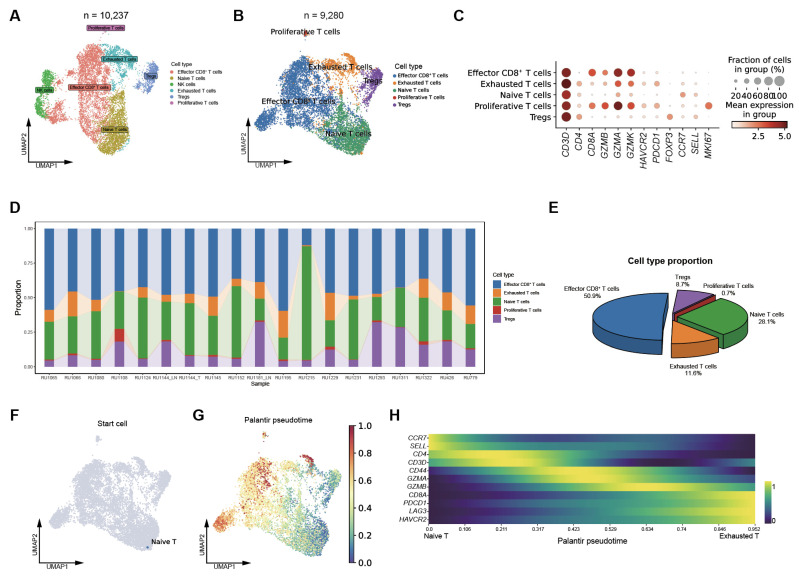
T cell heterogeneity and evolution in SCLC. (**A**) UMAP plot showing T and NK cell clusters, colored by cell type, across 20 SCLC samples; (**B**) UMAP visualization of T cell subsets, colored by cell type, across 20 SCLC samples; (**C**) Dot plot showing the expression patterns of representative marker genes across T cell subsets; (**D**) Bar plots depicting the proportions of individual T cell subsets across SCLC samples; (**E**) Pie chart summarizing the fractions of each T cell subset among total T cells; (**F**) UMAP plot showing the inferred starting cell for the trajectory analysis, corresponding to naïve T cell; (**G**) UMAP plot showing the inferred Palantir pseudotime trajectory of T cell evolution, revealing a continuum from naive T cells through effector CD8^+^ T cells toward an exhausted T-cell state; (**H**) Heatmap showing the smoothed expression dynamics of representative cytotoxicity- and dysfunction-associated genes along pseudotime.

To further dissect the T cell compartment, we re-clustered all T cells and identified a subpopulation of NK cells, which were subsequently excluded from downstream analyses (Figure 2A and Figure S1B). Based on representative marker gene expression, five major T cell subsets were identified: effector CD8^+^ T cells (*CD8A^+^*, *GZMB^+^*), exhausted T cells (*PDCD1^+^*, *HAVCR2^+^*), naïve T cells (*CCR7^+^*, *SELL^+^*), proliferative T cells (*MKI67^+^*), and regulatory T cells (Tregs) (*FOXP3^+^*) (Figure 2B,C). The proportions of these subsets varied substantially across samples, highlighting pronounced heterogeneity of T cells within the T cell compartment (Figure 2D,E). In most tumors, effector CD8^+^ T cells constituted more than half of the total T cell population. These cells expressed high levels of cytotoxic markers such as *GZMB*, while lacking exhaustion markers, including *PDCD1*, suggesting that they remain functionally active.

We next performed trajectory analysis to explore potential differentiation relationships among these T-cell subsets. Notably, we observed a continuous developmental trajectory originating from a naïve T cell state to an effector CD8^+^ T cell state, and ultimately progressing toward an exhausted T cell state (Figure 2F,G and Figure S1C). This trajectory was further supported by CellRank analysis (Figure 2H). Along the pseudotime trajectory, genes associated with cytotoxicity and dysfunction, such as *GZMA*, *GZMB*, *PDCD1*, and *HAVCR2*, were sequentially upregulated (Figure 2H), reflecting the transition of T cells from activation to terminal exhaustion. Collectively, these results delineate the transcriptional landscape and differentiation trajectories of T cells in SCLC, revealing substantial inter-tumoral heterogeneity in T cell composition and exhaustion dynamics that may contribute to variable responses to immunotherapy.

### 3.3. Identification of Epigenetic Regulators Negatively Correlated with Effector CD8^+^ T-Cell Infiltration and Immunotherapy Response in SCLC

**Figure 3:**
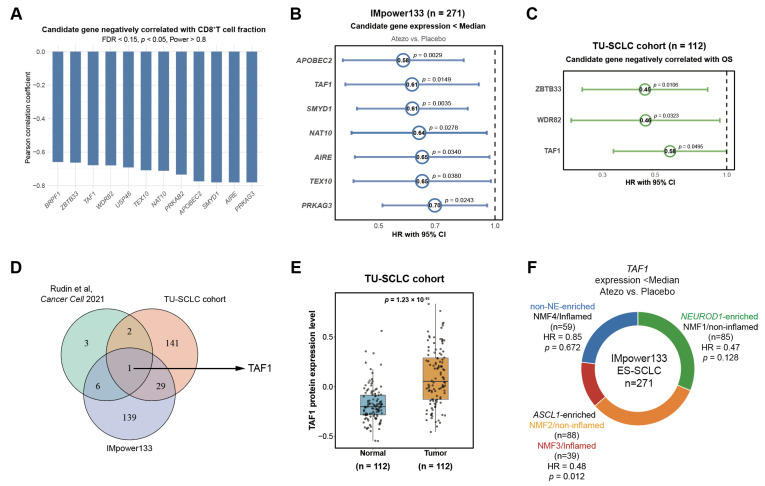
Epigenetic regulators negatively correlated with effector CD8^+^ T-cell infiltration and immunotherapy response in SCLC. (**A**) Bar plot showing the negative correlations between the expression levels of 12 epigenetic regulators in tumor cells and the proportion of effector CD8^+^ T-cell infiltration among total T cells in 20 SCLC samples from the Rudin et al. cohort; (**B**) Forest plots of univariable Cox regression analysis showing HRs (95% CIs) for OS of 7 genes with low expression associated with improved OS; atezolizumab + chemotherapy vs. chemotherapy alone; IMpower133 cohort (*n* = 271). (**C**) Forest plot depicting HRs with 95% CIs for OS of 3 candidates for which reduced protein expression is associated with better prognosis in the TU-SCLC proteomic cohort (*n* = 112); (**D**) Venn diagram showing the overlapping candidates across the scRNA-seq cohort [18], the IMpower133 transcriptomic cohort, and the TU-SCLC proteomic cohort; (**E**) Protein levels of four candidate epigenetic regulators in SCLC tumors versus matched adjacent normal tissues in the TU-SCLC proteomic cohort (*n* = 112); (**F**) Summary of the association between *TAF1* expression and OS in SCLC patients across different molecular subtypes.

To explore the epigenetic mechanisms regulating effector CD8^+^ T cell abundance, we systematically screened 796 epigenetic modifiers across three independent SCLC cohorts. In the Rudin et al. cohort, 12 genes were identified to be negatively correlated with effector CD8^+^ T cell infiltration (*R* < 0, *p* < 0.05, FDR < 0.15, Power > 0.8, Figure 3A, Table S1). In the IMpower133 cohort, univariate Cox regression analysis revealed 175 genes whose lower expression was associated with improved OS in patients receiving atezolizumab compared with placebo (HR < 1, *p* < 0.05, Table S2). Seven of these genes were among the 12 identified candidates in the Rudin et al. cohort (Figure 3B and Figure S2A). In the TU-SCLC proteomic cohort, using a best-cutoff approach for OS analysis, 173 genes with lower expression were correlated with improved patient survival (HR < 1, *p* < 0.05, Table S3). Three of these genes were among the 12 candidates in the Rudin et al. cohort (Figure 3C).

Through integrative analysis, we identified TAF1 as a common candidate across three cohorts (Figure 3D). In the IMpower133 cohort, multivariate Cox regression analysis further confirmed that patients with low *TAF1* expression displayed better OS (HR = 0.56, *p* = 0.0096, Figure S2B). TAF1 was also significantly upregulated in tumor tissues compared with matched adjacent normal tissues in the TU-SCLC cohort (Figure 3E). Given the pronounced molecular heterogeneity of SCLC, we next investigated whether this prognostic effect was restricted to specific molecular subtypes. Stratified survival analyses revealed that the adverse prognostic impact of high *TAF1* expression was most prominent in the *ASCL1*-high (SCLC-A) subtype, whereas it was not observed in the *NEUROD1*-high (SCLC-N) or non-NE subtypes (Figure 3F and Figure S2C–E). Together, these findings identify TAF1 as a potential regulator of immune evasion and a predictor of poor prognosis following immunotherapy in SCLC.

### 3.4. Validation of Negative Association Between TAF1 Expression and Effector CD8^+^ T-Cell Infiltration in SCLC

**Figure 4:**
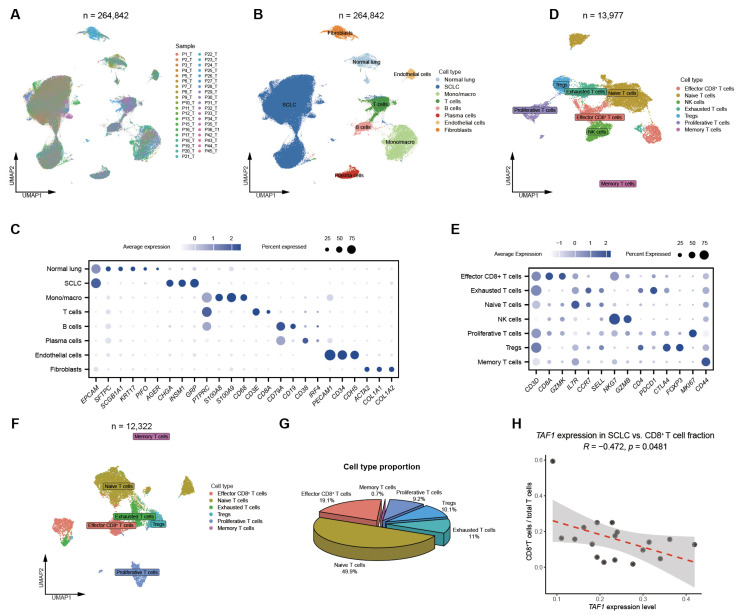
Negative association between *TAF1* expression and effector CD8^+^ T-cell infiltration in an independent SCLC cohort. (**A**) UMAP visualization of all cells from 39 SCLC samples, colored by sample identity, in the Wang et al. scRNA-seq dataset [20]; (**B**) UMAP visualization of all cells from 39 SCLC samples, colored by annotated cell types; (**C**) Dot plot showing the expression of representative lineage marker genes across major cell types; (**D**) UMAP plot showing T and NK cell subsets from 39 SCLC samples; (**E**) Dot plot showing representative gene expression patterns across T and NK cell subsets; (**F**) UMAP visualization of T cell subclusters; (**G**) Pie chart illustrating the proportions of individual T cell subsets among total T cells; (**H**) Correlation between the average *TAF1* expression level in the SCLC cells and the proportion of effector CD8^+^ T cells among total T cells in the Wang et al. cohort.

To further validate the association between *TAF1* expression and CD8^+^ T cell infiltration in SCLC, we re-analyzed an independent scRNA-seq dataset comprising 39 SCLC samples [20]. UMAP visualization recapitulated the major cellular populations and T-cell subsets identified in the Rudin et al. scRNA-seq dataset (Figure 4A–C). Re-clustering of all T cells also identified a subset of NK cells (Figure 4D,E), which were subsequently excluded from further analysis.

We then quantified the proportion of effector CD8^+^ T cells among total T cells across samples (Figure 4F,G and Figure S2F). Notably, *TAF1* expression in tumor cells was inversely correlated with the proportion of effector CD8^+^ T cells (*R* = −0.47, *p* = 0.0481; Figure 4H). We also assessed the relationship between TAF1 expression and regulatory T cells (Tregs) as well as macrophage subsets; however, no significant correlations were observed (Figure S2G–J). Together, these findings confirm a consistent and reproducible negative association between *TAF1* expression and CD8^+^ T cell infiltration across independent datasets, supporting a role for TAF1 as a potential epigenetic barrier to effective antitumor immunity in SCLC.

### 3.5. TAF1 Represses MHC-I Expression in SCLC

**Figure 5:**
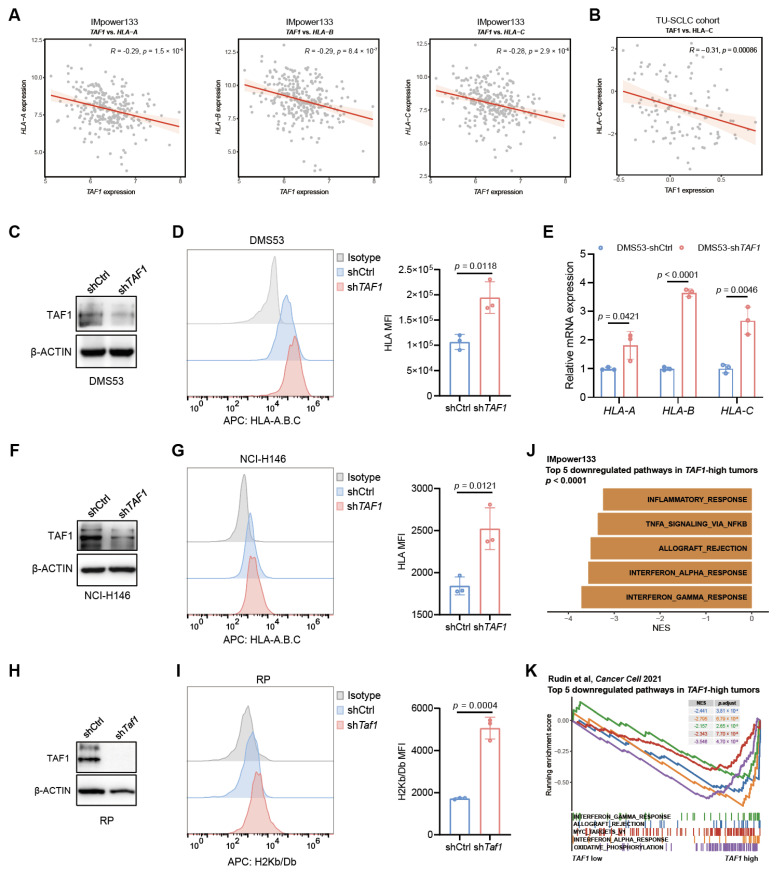
TAF1 represses MHC-I expression in SCLC. (**A**) Correlation between *TAF1* expression and MHC-I gene expression in the IMpower133 RNA-seq dataset; (**B**) Correlation between TAF1 expression and HLA-C protein levels in the TU-SCLC proteomic dataset; (**C**) Western blot analysis of *TAF1* expression in DMS53 cells with or without *TAF1* knockdown; β-actin was used as a loading control; (**D**) Flow cytometric analysis of HLA-A/B/C expression in DMS53-sh*TAF1* and DMS53-shCtrl cells; (**E**) qPCR analysis of representative HLA expression (*HLA-A*, *HLA-B*, *HLA-C*) in DMS53-sh*TAF1* versus DMS53-shCtrl cells; (**F**) Western blot analysis of TAF1 expression in NCI-H146 cells with or without *TAF1* knockdown; β-actin was used as a loading control; (**G**) Flow cytometric analysis of HLA-A/B/C expression in NCI-H146-sh*TAF1* and NCI-H146-shCtrl cells; (**H**) Western blot analysis of TAF1 expression in RP cells with or without *Taf1* knockdown; β-actin was used as a loading control; (**I**) Flow cytometric analysis of H2Kb/Db expression in RP-sh*Taf1* and RP-shCtrl cells; (**J**) Top five downregulated pathways in tumors with high *TAF1* expression compared to those with low *TAF1* expression in the IMpower133 cohort; (**K**) Top five downregulated pathways enriched in tumor cells with high TAF1 expression compared with low *TAF1* expression in Rudin et al. cohort.

To further investigate the immunoregulatory role of TAF1, we integrated multi-omics datasets across independent SCLC cohorts. In the IMpower133 cohort, *TAF1* expression was significantly negatively correlated with multiple MHC-I genes (*HLA-A*, *HLA-B*, *HLA-C*; Figure 5A) as well as antigen presentation machinery (APM) genes, including *TAP1* and *TAP2* (Figure S3A). Consistently, in the TU-SCLC cohort, TAF1 protein expression was inversely associated with the abundance of MHC-I and APM components, including HLA-C, TAP1, TAP2, and TAPBP (Figure 5B and Figure S3B). To experimentally validate these associations, we silenced TAF1 in three *ASCL1*-high SCLC cell lines: the human cell lines DMS53 and NCI-H146, and the murine RP line. Depletion of TAF1 resulted in a significant upregulation of cell-surface MHC-I in DMS53 cells at both the protein and mRNA levels (Figure 5C–E). Similarly, TAF1 depletion led to increased MHC-I protein expression in NCI-H146 and RP cells (Figure 5F–I). Together, these findings support a negative regulatory role of TAF1 in MHC-I expression in SCLC.

Additionally, gene set enrichment analysis (GSEA) of the IMpower133 transcriptomic data revealed that several immune-related pathways, including inflammatory response, TNFα signaling, and IFNα response, were significantly downregulated in SCLC tumors with high *TAF1* expression compared with those with low *TAF1* expression (Figure 5J). Similarly, in the Rudin et al. scRNA-seq cohort, *TAF1*-high tumor cells consistently showed suppression of IFN response genes, further supporting a role of TAF1 in inhibiting tumor cell-intrinsic immune signaling (Figure 5K and Figure S3C). Collectively, these findings demonstrate that TAF1 suppresses antigen presentation and dampens immune response signaling, thereby contributing to an immune-cold TME in SCLC.

### 3.6. TAF1 Depletion Inhibits Tumor Growth and Increases CD8^+^ T Cell Infiltration

**Figure 6:**
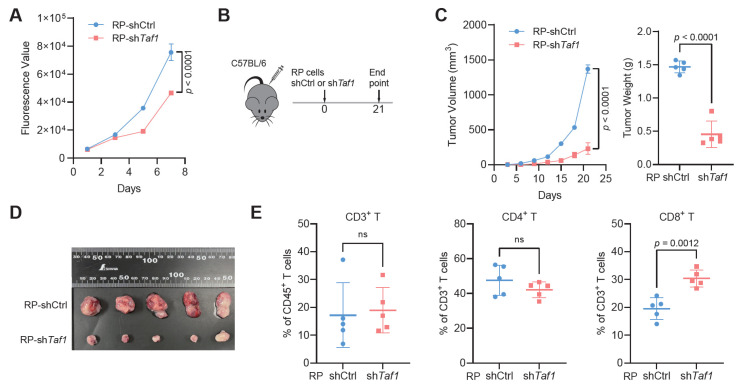
TAF1 depletion inhibits tumor growth and increases CD8*^+^* T cell infiltration. (**A**) CellTiter-Glo assay measuring proliferation of RP-shCtrl vs. RP-sh*Taf1* cells; (**B**) Schematic showing the allograft experiment design; (**C**) In vivo tumor growth curves of RP-shCtrl and RP-sh*Taf1* cells (left) in C57BL/6 mice, with tumor weights at the experiment endpoint (right; *n* = 5 mice/group); (**D**) Representative images of resected allograft tumors from RP-shCtrl and RP-sh*Taf1* groups (*n* = 5 tumors/group); (**E**) Flow-cytometric quantification of tumor-infiltrating T-cell subsets: CD3^+^ among CD45^+^ cells, CD4^+^ among CD3^+^ cells, and CD8^+^ among CD3^+^ cells. ns, not significant.

Finally, we assessed the functional impact of TAF1 depletion on SCLC progression and immune cell infiltration. *Taf1* depletion in RP cells significantly suppressed proliferation in vitro (Figure 6A). In a syngeneic allograft model, RP-sh*Taf1* cells exhibited significantly reduced tumor growth and lower endpoint tumor weights compared with control cells (Figure 6B–D). Flow cytometric analysis of tumor-infiltrating lymphocytes revealed no significant differences in the proportions of CD3^+^ T cells among CD45^+^ T cells or CD4^+^ T cells among CD3^+^ T cells. However, a significant increase in the proportion of CD8^+^ T cells within the CD3^+^ compartment was observed in RP-sh*Taf1* tumors (Figure 6E and Figure S3D). Together, these results demonstrate that *Taf1* knockdown not only attenuates tumor growth but also promotes CD8^+^ T cell infiltration.

## 4. Discussion

Most SCLCs show primary resistance to immunotherapy, yet the underlying mechanisms remain largely unknown. In this study, we identify TAF1 as a potential epigenetic regulator of immunogenicity and immunotherapy response in SCLC. Specifically, we demonstrate that genetic depletion of TAF1 upregulates MHC-I expression, suppresses tumor growth in SCLC models, and increases CD8^+^ T cell infiltration.

A well-documented mechanism of immune evasion in SCLC is the recurrent loss of MHC-I molecules [8,24]. This event, often driven by defective IFN signaling, impaired antigen-processing machinery, and epigenetic silencing, renders SCLC cells largely invisible to cytotoxic CD8^+^ T cells [25]. Notably, pharmacologic strategies such as DNA methyltransferase inhibitors can restore MHC-I expression and increase tumor immunogenicity [26,27]. Recent studies have shown that EZH2 inhibition reactivates the STING pathway and restores MHC-I expression, thereby enhancing SCLC immunogenicity and sensitivity to ICIs in SCLC [8]. Similarly, inhibition of LSD1 can re-activate MHC-I expression and enhance immunotherapy sensitivity in SCLC [9,10]. Our work extends this paradigm by demonstrating that genetic inhibition of TAF1 restores MHC-I in SCLC models and inhibits tumor growth. Therefore, our study expands the understanding of epigenetic regulation of immune escape in SCLC.

Identification of reliable biomarkers that predict immunotherapy response is urgently needed in SCLC. Here, we demonstrate that TAF1 represents a promising biomarker, with its expression negatively correlating with clinical outcomes in SCLC patients treated with chemoimmunotherapy. Interestingly, the prognostic and predictive value of *TAF1* expression was restricted to the SCLC-A subtype. This is likely due to the unique epigenetic and transcriptional landscape of SCLC-A subtype [28,29]. Given that TAF1 is a core component of the TFIID complex involved in transcription initiation, it is plausible that ASCL1-driven transcriptional programs may create a specific dependency on general transcriptional machinery components such as TAF1. Notably, the subtype-restricted vulnerability of SCLC to drug treatments has been widely recognized [3,4,30,31]. For example, the SCLC-A subtype is sensitive to BCL2 inhibitors and DLL3-targeting agents [3,31]. However, the precise mechanisms underlying TAF1 dependency in SCLC-A subtype remain to be determined in further studies.

This study has several limitations. First, the precise mechanisms by which TAF1 suppresses MHC-I expression remain to be fully elucidated. For example, whether TAF1 directly represses MHC-I transcription or regulates its expression through epigenetic mechanisms requires further investigation. Second, the functional role of TAF1 in regulating immunotherapy response in SCLC remains to be determined. Third, functional experiments were performed in a representative SCLC-A model, and validation across other subtypes will be essential to confirm the generalizability of our findings. Finally, prospective clinical validation of TAF1 as a predictive biomarker is warranted.

## 5. Conclusions

In conclusion, our findings identify TAF1 as a potential epigenetic suppressor of MHC-I expression in SCLC. This work positions TAF1 as both a candidate biomarker of immunotherapy response and a promising therapeutic target for enhancing immunotherapy efficacy in SCLC.

## Data Availability

The R/Python scripts used in the study have been shared on GitHub (https://github.com/yexian123/TAF1-SCLC, accessed on 15 April 2026). All data generated during this study are included in this article and its Supplementary Materials. Further inquiries can be directed to the corresponding authors.

## Supplementary Materials

The following supporting information can be downloaded at: https://www.mdpi.com/article/10.3390/biomedicines14050973/s1, Figure S1: (A) UMAP plots showing expression levels of representative marker genes across major cell types; the scale bar indicates relative expression levels; (B) Heatmap showing the expression of representative marker genes across T and NK cell clusters; the scale bar indicates relative expression levels; (C) Terminal state probabilities for each T cell subset predicted by CellRank. Figure S2: (A) Forest plots of univariable Cox regression analysis showing HRs (95% CIs) for OS of 7 genes with high expression; atezolizumab + chemotherapy vs. chemotherapy alone; IMpower133 cohort (*n* = 271). No significant differences in HR were observed between treatment arms; (B) Multivariate Cox regression adjusted for age, sex, metastatic sites, brain/liver metastasis, ECOG, LDH, bTMB, and PD-L1. HR < 1 favors atezolizumab. *TAF1*-low patients showed significant OS benefit with atezolizumab, while TAF1-high patients did not; (C–E) Subtype-specific survival analyses demonstrating that the adverse prognostic impact of high *TAF1* expression is confined to *ASCL1*-high tumors (C), but not observed in *NEUROD1*-high (D) or non-NE-enriched (E) subtypes; (F) Bar plot showing the proportions of individual T cell subset across samples in the Wang et al. scRNA-seq dataset; (G) Correlation between the average TAF1 expression level in the SCLC cluster and the proportion of Tregs among total T cells in the Wang et al. cohort; (H) UMAP visualization of myeloid cell subclusters in the Wang et al. cohort; (I) Dot plot showing the expression of canonical marker genes used for myeloid cell annotation in the Wang et al. cohort; the scale bar indicates average gene expression; dot sizes represent the proportion of cells expressing each gene; (J) Correlation between the average *TAF1* expression level in the SCLC clusters and myeloid subset proportions. Red/blue bars indicate positive/negative correlations. Figure S3: (A) Correlation between *TAF1* expression and *TAP1*, *TAP2* gene expression in the IMpower133 RNA-seq dataset; (B) Correlation between TAF1 expression and TAP1, TAP2, TAPBP protein levels in the TU-SCLC proteomic dataset; (C) UMAP plot showing *TAF1* expression across tumor cell clusters from 20 SCLC tumors in the Rudin et al. cohort; (D) Flow cytometry flowchart of subcutaneous tumors in C57BL/6 mice. Table S1: Epigenetic-related genes correlated with effector CD8^+^ T cell proportion in SCLC (Rudin et al. cohort). Table S2: Cox regression analysis of epigenetic-related genes with overall survival in IMpower133 cohort stratified by atezolizumab treatment. Table S3: Cox regression analysis of epigenetic-related genes with overall survival in the TU-cohort. Table S4: Summary of qRT-PCR primers.

## Abbreviations

The following abbreviations are used in this manuscript:

SCLC	Small cell lung cancer
scRNA-seq	Single-cell RNA sequencing
NE	Neuroendocrine
ICIs	Immune checkpoint inhibitors
OS	Overall survival
TAF1	TATA-box binding protein-associated factor 1
SPF	Specific pathogen-free
MHC-I	Major histocompatibility complex class I
APM	Antigen presentation machinery
GSEA	Gene set enrichment analysis
TME	Tumor microenvironment
HR	Hazard ratio
CI	Confidence interval
