# Supplementary figures and images for: TAF1 Suppresses MHC-I Expression and Correlates with Poor Immunotherapy Response in Small Cell Lung Cancer
Source: Biomedicines. 2026 Apr 23;14(5):973. doi: 10.3390/biomedicines14050973 (PMC13203750; doi:10.3390/biomedicines14050973)

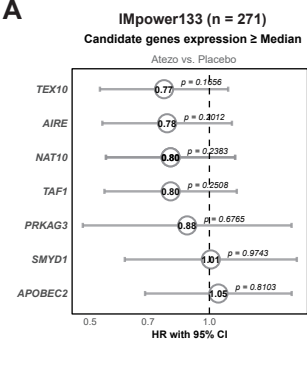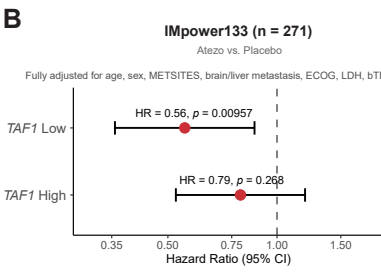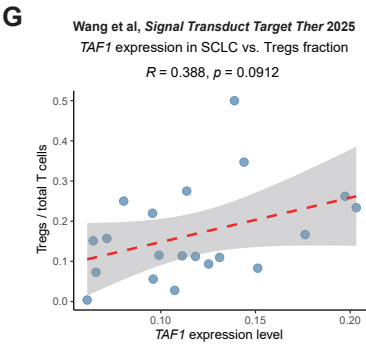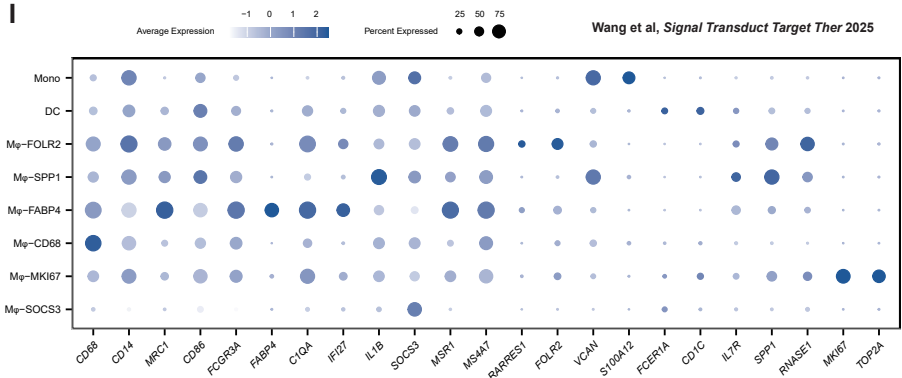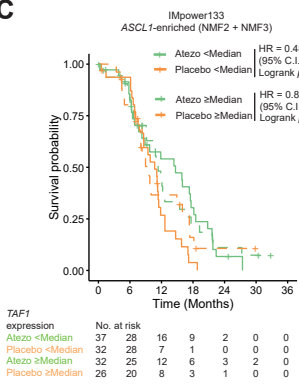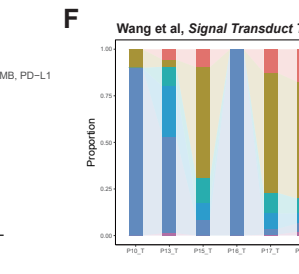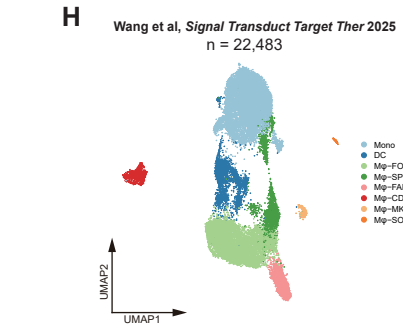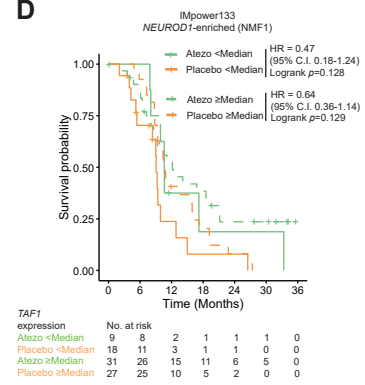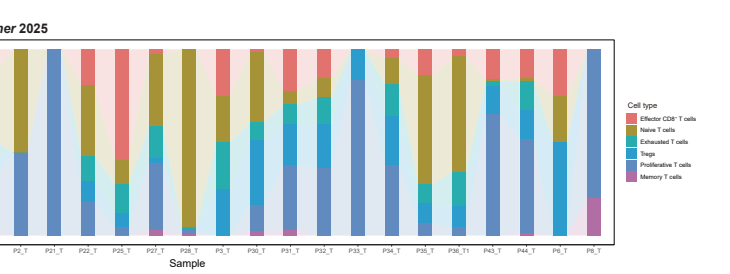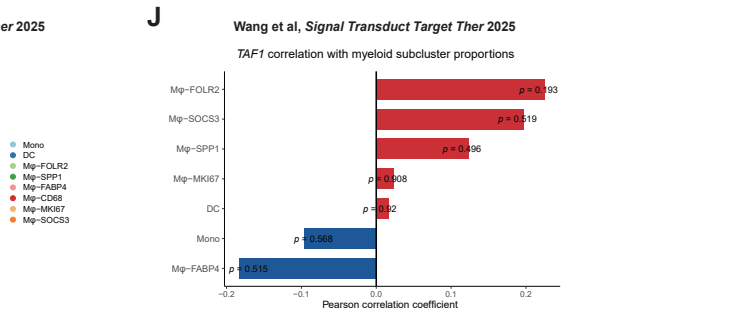

Supplement: Supplementary file 1 [file biomedicines-14-00973-s001.zip › Figure S2.pdf]

**A**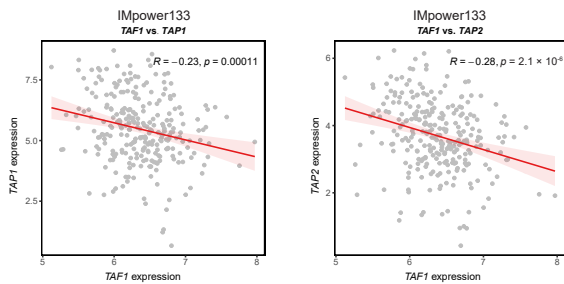**C**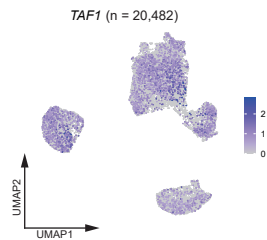**B**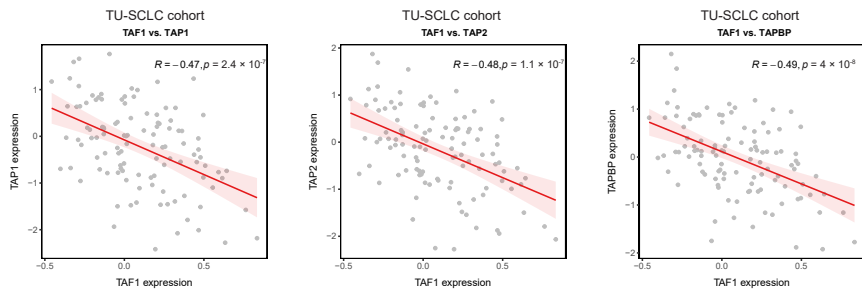**D**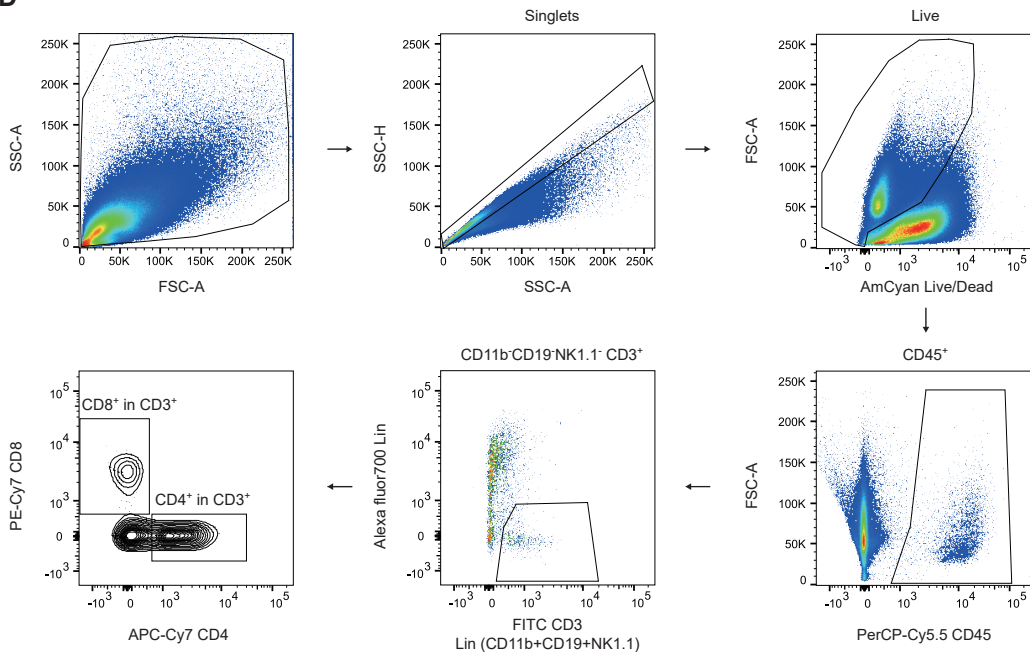

Supplement: Supplementary file 1 [file biomedicines-14-00973-s001.zip › Figure S3.pdf]
