# Supplementary material for: TAF1 Suppresses MHC-I Expression and Correlates with Poor Immunotherapy Response in Small Cell Lung Cancer
Source: Biomedicines. 2026 Apr 23;14(5):973. doi: 10.3390/biomedicines14050973 (PMC13203750; doi:10.3390/biomedicines14050973)
Supplement: Supplementary file 1 [file Figure S1.pdf › Figure S1.pdf]

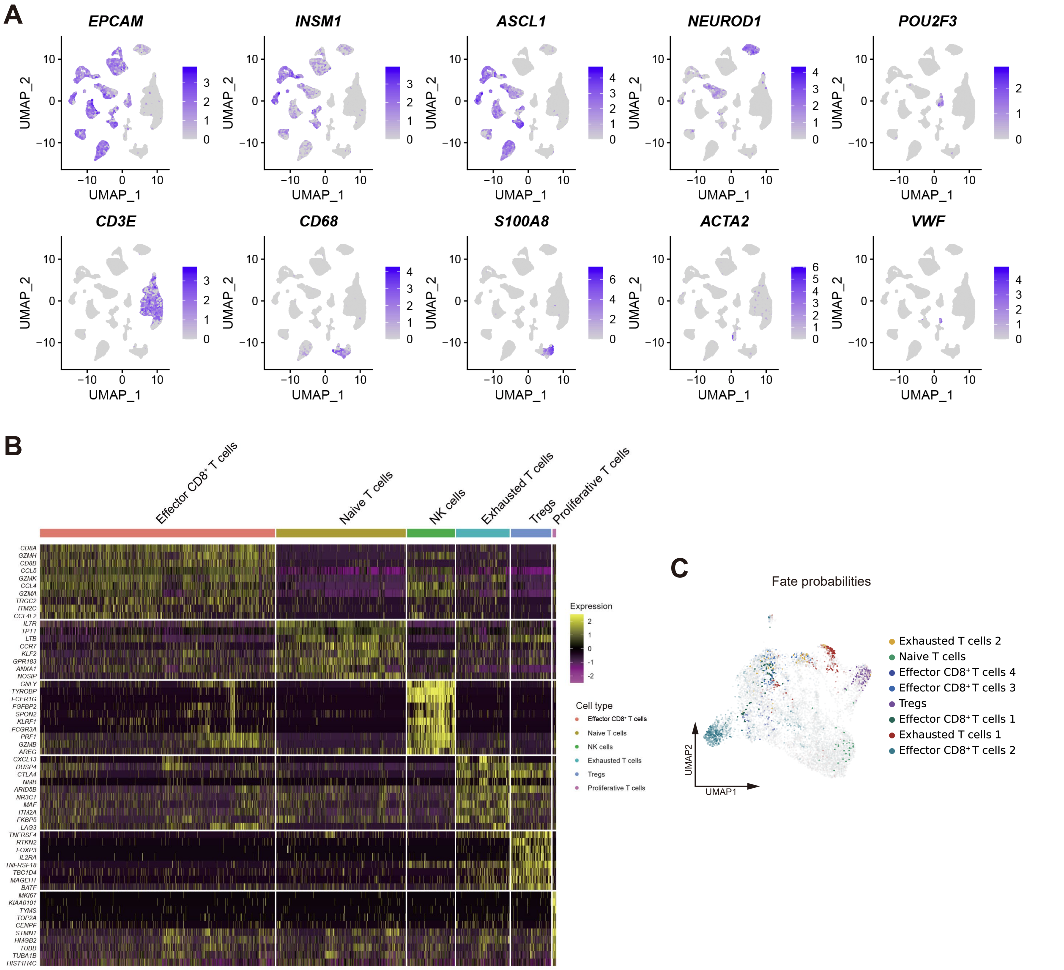
A
EPCAM
INSM1
ASCL1
NEUROD1
POU2F3
CD3E
CD68
S100A8
ACTA2
VWF
UMAP_1
UMAP_2
B
Effector CD8⁺ T cells
Naive T cells
NK cells
Exhausted T cells
Tregs
Proliferative T cells
CD8A
GZMH
CD8B
CCL5
GZMK
CCL4
GZMA
TRGC2
ITM2C
CCL4L2
IL7R
TPT1
LTB
CCR7
KLF2
GPR183
ANXA1
NOSIP
GNLY
TYROBP
FCER1G
FGFBP2
SPON2
KLRF1
FCGR3A
PRF1
GZMB
AREG
CXCL13
DUSP4
CTLA4
NMB
ARID5B
NR3C1
MAF
ITM2A
FKBP5
LAG3
TNFRSF4
RTKN2
FOXP3
IL2RA
TNFRSF18
TBC1D4
MAGEH1
BATF
MKI67
KIAA0101
TYMS
TOP2A
CENPF
STMN1
HMGB2
TUBB
TUBA1B
HIST1H4C
Expression
Cell type
Effector CD8⁺ T cells
Naive T cells
NK cells
Exhausted T cells
Tregs
Proliferative T cells
C
Fate probabilities
Exhausted T cells 2
Naive T cells
Effector CD8⁺ T cells 4
Effector CD8⁺ T cells 3
Tregs
Effector CD8⁺ T cells 1
Exhausted T cells 1
Effector CD8⁺ T cells 2
UMAP1
UMAP2
